# The Disordered Dehydrin and Its Role in Plant Protection: A Biochemical Perspective

**DOI:** 10.3390/biom12020294

**Published:** 2022-02-11

**Authors:** Margaret A. Smith, Steffen P. Graether

**Affiliations:** 1 Department of Molecular and Cellular Biology, University of Guelph, Guelph, ON N1G 2W1, Canada; msmith57@uoguelph.ca; 2 Department of Molecular and Cellular Biology and Graduate Program in Bioinformatics, University of Guelph, Guelph, ON N1G 2W1, Canada

**Keywords:** dehydrins, abiotic stress, cryoprotection, dehydration, intrinsically disordered, structure

## Abstract

Dehydrins are intrinsically disordered proteins composed of several well conserved sequence motifs known as the Y-, S-, F-, and K-segments, the latter of which is a defining feature of all dehydrins. These segments are interspersed by regions of low sequence conservation and are organized modularly, which results in seven different architectures: K_n_, SK_n_, Y_n_SK_n_, Y_n_K_n_, K_n_S, F_n_K and F_n_SK_n_. Dehydrins are expressed ubiquitously throughout the plant kingdom during periods of low intracellular water content, and are capable of improving desiccation tolerance in plants. In vitro evidence of dehydrins shows that they are involved in the protection of membranes, proteins and DNA from abiotic stresses. However, the molecular mechanisms by which these actions are achieved are as of yet somewhat unclear. With regards to macromolecule cryoprotection, there is evidence to suggest that a molecular shield-like protective effect is primarily influenced by the hydrodynamic radius of the dehydrin and to a lesser extent by the charge and hydrophobicity. The interaction between dehydrins and membranes is thought to be a surface-level, charge-based interaction that may help to lower the transition temperature, allowing membranes to maintain fluidity at low temperatures and preventing membrane fusion. In addition, dehydrins are able to protect DNA from damage, showing that these abiotic stress protection proteins have multiple roles.

## 1. Introduction

When subjected to unfavorable abiotic stresses, plants are unable to move away from their surroundings and must instead rely on defense mechanisms for survival. One of the most prevalent challenges plants faces is desiccation. On a molecular level, the absence of water reduces the hydrophobic effect, which can lead to protein denaturation and the transition of membranes from the lamellar to the reversed hexagonal (H_II_) phase [1]. Additionally, water loss brings plasma and endosomal membranes into closer proximity, allowing lipids to be exchanged, which alters membrane permeability [1]. This leads to a chain reaction of ion influx, an increase in reactive oxygen species (ROS), and oxidative damage to lipids, proteins and nucleic acids, any of which could lead to the death of the plant.

One of the molecular mechanisms with which a plant responds to abiotic stress is the expression of proteins in the late embryogenesis abundant (LEA) gene family. These were originally discovered during late embryogenesis, a period marked by desiccation, in *Gossypium hirsutum* seeds [2]; subsequently, LEA have been found to be expressed in desiccated vegetative tissue as well [3]. This is true of all organisms within the plant kingdom, including non-vascular plants [4,5]; however, green algae have significantly fewer LEA proteins within its genome [6].

The upregulated expression of an LEA protein generally occurs in response to at least one of three abiotic stresses, drought, cold and osmotic stress, all of which lead to the depletion of intracellular water [7]. Additionally, abscisic acid (ABA), a hormone involved in the maturation of embryos, is involved in the upregulation of some LEA proteins [2]. Elevated levels of ABA and LEA proteins coincide during late embryogenesis [2]; however, exogenous application of ABA can lead to an increase in LEA protein in vegetative tissue as well [8]. Plant genomes often encode several LEA protein families, which are not necessarily upregulated in response to the same stresses and can be unique to specific stages of growth [6,9,10]. Nonetheless, the expression of these proteins tends to coincide with increased desiccation tolerance. This has been demonstrated by transgenic plants over many studies, and can be seen manifested in reduced electrolyte leakage and lipid peroxidation [11,12,13,14,15,16] as well as in improved growth and survival under conditions that represent desiccation [12,13,17].

For example, transgenic *Oryza sativa* L. grown with 200 mM sodium chloride and expressing a dehydrin protein from wheat , experienced a 1.1–1.5-fold increase in electrolyte leakage between the third and seventh day. In contrast, leakage increased 3.4-fold in the control plants [12]. Similarly, six-week old transgenic tobacco expressing the maize dehydrin ZmDHN2b showed a 2.2–2.4-fold increase in electrolyte leakage after spending 24 h at 4 °C, whereas the control plants showed a 3.1-fold increase [15]. Plant growth and germination is improved under conditions of stress in plants overexpressing LEA proteins. Cheng et al. [12] found that transgenic rice was ~120–130% the height of the control plants, and that the fresh and dry shoot weight improved significantly under both osmotic and dehydration stress. The dehydrin-expressing transgenic tobacco of Hara et al. [13] had a greater increase in fresh weight over the course of fifteen days at 15 °C, as well as improved seed germination characteristics. The GD_50_ (days required for 50% of seeds to germinate) at 15 °C was 4.1 days for the transgenic dehydrin plants and 6.4 days for the transgenic control plants [13]. A summary of these effects and those observed in other plants is shown in Table 1.

The mechanisms by which this improved desiccation tolerance is achieved are as of yet unclear, although several potential roles have been identified in vitro, including membrane stabilization, ion and ROS sequestration, and macromolecule cryoprotection. However, the mechanisms underlying these potential modes of action are difficult to elucidate on a molecular level, in large part because of the disorder and sequence diversity inherent to members of the LEA protein family. In the remainder of this review, we will mainly focus on one molecular mechanism that is correlated with desiccation stress in the best studied LEA proteins: the dehydration-induced proteins, or dehydrins. Before discussing these mechanisms, we examine the sequence and architecture of the dehydrins.

## 2. Dehydrin Sequence and Architecture

Dehydrin is the name for the group 2 LEAs, as defined by Dure et al. [18], mainly characterized by the conserved motifs known as Y-, S-, F- and K-segments [7,19]. Each segment is named after conserved residues within the motif; the Y-segment has a central aromatic residue, often Tyr, the S-segment has 5–7 Ser residues in a row, the F-segment contains one or two Phe residues, and the K-segment is Lys-rich. By definition, a dehydrin must contain at least one K-segment; this definition arises because they are defined by their sequence, rather than by their specific function. In the literature, the K-segment is often described by the canonical sequence EKKGIMDKIKEKLPG, which can be misinterpreted as being a fairly conserved motif. A detailed analysis of the K-segment suggests it can be more accurately described as (XKXGXX(D/E)KIK(D/E)KXPG) [20]. In this representation, the center of the segment is defined by a highly conserved (Asp or Glu)-Lys-Ile-Lys-(Asp or Glu)-Lys sequence, which highlights the presence of three Lys residues and hence the name of K-segment. The conservation of the Asp/Glu and Ile residues shows another pattern typically found in the K-segment, that is, the conservation of the biochemical properties at a specific residue position, especially charge and hydrophobicity. The function of the K-segment is its role in protecting membranes from cold-stress damage as well as possibly in enzyme cryoprotection, which is described in greater detail below. The F-segment is named after the presence of two Phe residues near the middle of the motif [19]. An analysis of 426 dehydrins suggests that this motif could be shown as [EXXDRGXFDFX(G/K)], where the seventh and tenth position are hydrophobic amino acids [21]. A detailed function for this motif has yet to be described, though it may have a role in enzyme cryoprotection (described below).

The Y-segment, like the K-segment, is often described by a single sequence: DEYGNP, but could be better described as [D(D/E)(Y/H/F)GNPX], where the X is often a hydrophobic amino acid. While the motif is named after the central Tyr residue, it is one of the less conserved residue positions; often another aromatic residue is found at this position, that is, His or Phe. It is important to note that Trp, an aromatic but order-promoting amino acid, is never found here. Previously, sequence similarity between the Y-segment and the nucleotide-binding domain of chaperones led to speculation about the Y-segment potentially acting as a nucleotide-binding site [7]. However, direct testing by NMR spectroscopy showed that the *Vitis riparia* dehydrin YSK_2_ does not bind ATP [22]. The biochemical role of the Y-segment has not yet been determined, although other studies have provided clues. It seems likely that ancestral dehydrins prior to the division of the angiosperms and gymnosperms were F-segment-containing dehydrins, because 25% of gymnosperm dehydrins possess F-segments and none have Y-segments. This suggests that the Y-segment may serve some purpose in coated seeds [21].

Lastly, the S-segment is defined by the presence of 5–7 Ser residues in a row. Further analysis of the dehydrins has shown that the motif contains other conserved residues, and can be described as [LHR(S/T)GS_4–6_(S/D/E)(D/E)_3_]. Not surprisingly, the S-segment is a target for phosphorylation, likely by an SNF1-related protein kinase 2 (SnRK2) [23]. This post-translational modification has been shown to have two effects on dehydrins. The first is that the phosphorylated S-segment binds to calcium ions, possibly buffering their intracellular concentration [24]. The other is that phosphorylation has been shown to increase localization of the dehydrin from the cytosol to the nucleus [25].

Separating these conserved motifs are the ϕ-segments, which while poorly conserved are rich in Gly (17.5%), Glu (11%) and Thr (10.7%), and very low in Trp (0.05%), Cys (0.4%) and other bulky hydrophobic residues [20]. Despite the lack of conservation, the ϕ-segments are not random, and MEME analysis has been used to determine the existence of a GT-rich motif wherein Gly and Thr occupy 41% and 39% of positions respectively [20]. These motifs are most commonly positioned between Y- and S-segments (76.7%) and sometimes between K-segments (40.6%), and have been found in all Y_n_SK_n_ and 82% of Y_n_K_n_ dehydrins [20]. While the length of the ϕ-segments is variable, ranging from 1 to 500 residues, they are mostly <100 residues long. This variability suggests that these segments may not have a specific function; instead, it may be that their high flexibility allows the conserved motifs to be positioned as necessary to carry out the function of the motifs.

In dehydrins, these motifs organize to create architectures into which dehydrins are subdivided, namely, K_n_, SK_n_, Y_n_SK_n_, Y_n_K_n_ and K_n_S, where ‘n’ indicates the number of times each motif appears [7]. With the relatively recent discovery of the F-segment, the list of architectures has been extended to seven in order to include F_n_K_n_ and F_n_SK_n_ [19]. Generally, the Y- or F-segments are N-terminal to all other motifs, while the S-segment is N-terminal to the first K-segment, with the exception of the K_n_S architecture [7]. The S-segment only ever appears once within a dehydrin, whereas the Y-segment tends to appear one to three times, though a Y_11_K dehydrin was reported in *P. patens* [26]. The F-segment can appear up to three times in F_n_SK_n_ and F_n_K_n_ dehydrins, not unlike the Y-segment; while dissimilar in terms of sequence, the F- and Y-segments do share central aromatic residues, something that is otherwise rare in dehydrin sequences [19,27]. The K-segment is the most variable in terms of frequency, ranging from one to thirteen copies and most often appearing twice [20,28].

There are a few known dehydrins that fall outside of these seven architectures [19,20]. These are an SK_3_S dehydrin from *Stellaria longipes* [29], an SK_2_YKYK dehydrin from *Cerastium arcticum* [30], a K_4_SK_2_ dehydrin from *Picea abies* (ABS58630.1), and a Y_2_KY_2_KY dehydrin from *Juglans regia* (AGJ94410.1) [19]. However, it is possible that the genes encoding these dehydrins were misannotated, and would therefore first need to be confirmed by proteomics.

## 3. Evolution of Dehydrins

The evolutionary relationship between the different dehydrin architectures has not been fully elucidated to date. It is difficult to do so because of the lack of conservation in the ϕ-segments and the variable number of conserved motifs, which makes obtaining the accurate multiple sequence alignments necessary for phylogenetic studies very challenging. However, several studies have analyzed the genomes of various plants (rice, wheat, kiwi, locoweed, potatoes) with the intention of identifying the dehydrins and creating phylogenetic trees in order to better understand their evolution within that plant. These studies often conclude that tandem and/or segmental duplication are major factors in the diversification of the dehydrin family [31,32,33,34,35]. One of the more comprehensive family-oriented studies focused on the *Pinaceae* family, where the authors identified the N1-segment, which is now known as the F-segment [36]. The results of the phylogenetic analysis found that the *Pinaceae* dehydrins are split into two groups, one containing the FSK_n_ dehydrins and the other containing both FK_n_ and K_n_ dehydrins. Within the latter group, the FK_n_ and K_n_ dehydrins split into two clades each, which suggests a parallel loss of sequence motifs. When angiosperms were included in the phylogenetic tree, they formed their own clades separate from those of *Pinaceae*. Consequently, the authors predict that a whole-genome duplication event may have occurred after the split of angiosperms and gymnosperms and prior to the divergence of monocots and dicots [36]. During this time there may have been further duplications and the parallel gain and loss of sequence motifs, including the loss of the F-segment and acquisition of the Y-segment in some angiosperms.

In another study, an examination of synteny communities in the dehydrins of 60 angiosperm species found that of the 365 dehydrins studied, 62.2% fell into one of twelve synteny communities [37]. Two of the synteny communities were larger than the others: group 1, composed mostly of Y_n_SK_n_ dehydrins, was most often expressed during seed development, while group 2, composed mainly of (F_n_)SK_n_ dehydrins, was expressed primarily in response to abiotic stress. Neither of these groups was limited to either monocots or dicots, leading the authors to speculate that a duplication event happened before their divergence. Different regulatory elements could have been acquired following the duplication event, leading the groups to evolve to be expressed under different conditions [37].

Our analysis of a large number of plant genomes of the Brassicaceae family revealed several patterns [21]. The first subtree contained all the Y_n_K_n_ (Y_3_K) and nine Y_n_SK_n_ (Y_3_SK_2_) dehydrins from the *Brassicaceae* family. Within the species of this family, both genes were often present on different chromosomes, whereas close relatives of the Brassicaceae family only ever had one Y_n_SK_n_-type orthologue of either a Y_3_K or Y_3_SK_2_ dehydrin from the subtree and never both. The genes encoding these proteins were well conserved, suggesting a duplication event. The Y-segments of these dehydrins aligned well, as did the K-segment of the Y_3_K dehydrins with the C-terminal K-segment of the Y_3_SK_2_ dehydrins. These dehydrins were found to have similar expression patterns in *Arabidopsis,* although the Y_3_SK_2_ dehydrins seemed to be more upregulated. Taken together, this suggests that a whole-genome duplication likely occurred after *Carica papaya* split from the *Brassicaceae* family and before the arrival of the *Brassicaceae* lineage, and that the Y_3_K dehydrins may be on their way to pseudogenization [21].

The second subtree contained all but one of the K_n_ from *Brassicaceae*, as well as six Y_n_SK_n_ (YSK_2_) dehydrins and an SK_2_ dehydrin. The K_n_ dehydrins were all K_6_, with the exception of one K_5_ dehydrin. The K_6_ and YSK_2_ dehydrins are adjacent to each other in the genomes, which suggests that the K_n_ dehydrin arose by tandem duplication from the YSK_2_ gene. This is supported by the observation that the K-segments of the K_6_ dehydrins and the C-terminal K-segment of the YSK_2_ dehydrins showed high sequence similarity. The expression patterns of these dehydrins differed in that greater upregulation was observed for the K_6_ dehydrins in response to abiotic stress, whereas the YSK_2_ dehydrins were largely expressed in the late seed stages. A lack of orthologs in other closely related species as well as the absence of K_6_ in *Brassica rapa* and *Eutrema salsugineum* points to YSK_2_ being present prior to the duplication event that likely occurred after the separation of *Arabidopsis* from *B. rapa* and *E. salsugineum* and before the *Arabidopsis* and *Capsella* lineages split. The Y- and S-segments would have to have been lost and the K-segment repeated in order to obtain K_6_; however, given the differences in expression patterns it would seem that in the process K_6_ has acquired a unique function separate from that of YSK_2_ [21].

## 4. Intrinsic Disorder and Structure of Dehydrins

Many members of the LEA protein family are intrinsically disordered proteins (IDPs), which is to say that they possess very little secondary structure, no defined tertiary structure, and are highly flexible and extended when alone in solution. IDPs will rapidly cycle through a series of conformers that are limited mainly by backbone constraints and electrostatic repulsion; this is largely attributed to a lack of hydrophobic residues and an abundance of glycine and polar and charged residues [38]. The flexibility of IDPs sets them apart from structured proteins in several ways. Notably, the native state of a random coil IDP has no defined structure and consequently cannot lose integrity under conditions where folded proteins would denature [39], an attribute that is likely critical for stress proteins such as dehydrins. IDPs lack predefined binding sites and are more likely to participate in promiscuous interactions with multiple partners, wherein they assume a different conformation for each target.

X-ray crystallography cannot be used to study dehydrins because the absence of static structure prevents the repeating interactions necessary for crystallization [40]. As such, circular dichroism (CD) and NMR have been used to examine their structure. CD studies have resulted in similar observations for all dehydrins, with the spectra showing a minimum around 200 nm and low ellipticity above 210 nm. This correlates with the random coil that dominates the structure. Sometimes a weaker peak at 222 nm is present, indicating the presence of weak α-helicity. Previously, a K_2_ dehydrin from *Vitis riparia* (also known as VrDHN1) was found to have a <5% α-helical character localized to the central residues of the K-segments [41]. CD reports on the global structure of a protein, and cannot determine whether the 5% helicity represents 5% of the sequence being completely helical, 5% of the whole protein being helical at any given time, or something in between. Residue-specific information can be obtained from advanced NMR experiments. NMR data coupled with the use of the secondary structure propensity (SSP) analysis program showed that it is the K-segments that have a weak helical character [42].

CD experiments are excellent tools to study changes in dehydrin structure under conditions meant to mimic the crowding experienced in a dehydrated cell. Ordered proteins often favor a more compact/stable conformation as competition for space increases; however, the dehydrins studied were not greatly affected by the presence of crowding agents (Ficoll and glycerol) and were shown to retain their disorder and flexibility [43].

The intrinsic disorder of dehydrins has an important experimental effect. Among dehydrins, the apparent mass of an IDP (as determined by size exclusion chromatography or SDS-PAGE) is almost always an overestimate of the actual mass. In the case of size exclusion chromatography this is due to a lack of compaction that increases the hydrodynamic radius of these proteins relative to ordered proteins, while with SDS-PAGE the absence of hydrophobic residues results in less SDS binding to the protein, affecting its net charge [44]. In both cases, the lower mobility of the protein results in a higher predicted mass. This can easily be twice the mass predicted by sequence alone, which has led some researchers to erroneously propose that a particular dehydrin is a dimer. Therefore, molecular weights reported in early papers in the field where the value is only based on SDS-PAGE or size-exclusion chromatography should be interpreted with caution.

Nonetheless, other studies have used other techniques to show that some dehydrins are able to form oligomers. A split-ubiquitin yeast two hybrid (YTH) system was used to examine the dimerization of a dehydrin from *Opuntia streptacantha* (OpsDHN1), with β-galactosidase activity being used as a quantitative measure of binding strength. Upon deletion of the C-terminal K-segment of OpsDHN1 (making it an SK_2_) dimerization with the full-length SK_3_ was attenuated, while dimerization with the other truncated SK_2_ dehydrins was completely abolished. Additionally, OpsDHN1 was truncated to only include the S-segment and the poly-Lys segment found to be C-terminal to the S-segment; this construct was unable to associate with any form of OpsDHN1. Additionally, the His-rich segment was deleted, and this construct (OpsDHN1-∆His) was unable to dimerize with itself and proved to have an even weaker interaction with SK_3_ than the SK_2_ construct did [45]. However, in a follow-up study using a bimolecular fluorescence complementation assay (BiFC assay), OpsDHN1-∆His was found to dimerize with itself [46]. The authors attribute this to shortcomings in the split-ubiquitin YTH system, such as the possibility that the split-ubiquitin impeded some important interactions [46]. In a similar experiment, homo- and heterodimers were found to form between several *Arabidopsis thaliana* dehydrins (AtCOR47, AtERD10 and AtRAB18) as well as with OpsDHN1. None of these interactions required the His-rich segment, although the K-segment may have been involved [47]. The His-rich motif did prove to be necessary for nuclear localization [46]; however, it is as of yet unclear whether or not dimerization is required in order to reach the nucleus.

## 5. Membrane Binding

IDPs can gain structure in the presence of their cognate ligands, something which has been shown for dehydrins in the presence of a membrane. This is not surprising, as desiccation can have a negative effect on membrane integrity by inducing the transition from a lamellar phase to a reversed hexagonal phase (H_II_ phase) as well as by shortening the distance between plasma and endosomal membranes [1]. This can lead to membrane exchange, which may permanently compromise permeability and lead to unwanted ion influx, which in turn leads to increased oxidative damage to lipids, proteins, and DNA [48].

Initial evidence for a role in membrane protection came from several studies which used CD to show that the α-helical character of dehydrins could noticeably increase in the presence of SDS micelles, a membrane mimetic [49,50,51]. Later, a YSK_2_ dehydrin from *Zea maize*, ZmDHN1, was found to bind anionic small unilamellar vesicles (SUVs), with the exception of those made of phosphatidylinositol (PI) [52]. This was accompanied by an increase in the α-helical character of the K-segments, which were thought to be amphipathic α-helices [53]. These helices have been further confirmed using NMR to obtain residue specific information. The K-segments of the minimal K_2_ dehydrin from *Vitis riparia*, a truncated derivative of a YSK_2_ dehydrin [54], were determined to have some probability (ranging from 0.1–1.0) of α-helicity in the presence of SDS micelles, as determined by the δ2D program [55]. The probability was distributed such that residues in the center of the K-segments (the Lys-Ile-Lys region) had the greatest chance of being helical, decreasing towards the termini [55]. An interesting feature of these transient helices is that the Lys residues are predicted to sit at the interface between the hydrophobic and hydrophilic sides of the helix [52,55,56]. Clarke et al. [55] proposed that this may allow the hydrophobic face to bury into the acyl chains while the hydrocarbons of lysine ‘snorkel’ up to the surface [57], allowing the cationic amine to interact with anionic lipid headgroups. A study by Eriksson et al. [56] showed that the residues flanking the K-segment are important for membrane binding in certain dehydrins. They examined the dehydrin Lti30, which has histidine dipeptides flanking five of its six K-segments. When these dipeptides were deprotonated, binding was abolished. While the His residues are not a part of the transient helix, they do appear to position themselves in such a way as to interact with anionic headgroups [56]. Molecular dynamic simulations with the Lti30 His-K-segments found that there is a positive correlation between the length of the helix, the number of contacts made with lipids, and the depth at which the helix sits within the membrane [58]. Polarization transfer solid-state NMR was subsequently used to determine that the protein mostly interacts with the surface of the liposome, reducing the mobility of the acyl chains nearest the headgroups while having little effect on those found deep within the bilayer [59]. The lipid glycerol backbone has its mobility reduced, especially the carbon nearest the phosphate group. This is consistent with dehydrin binding to the anionic lipid headgroups, much of which is dependent on electrostatic interactions with the cationic residues within the amphipathic helix and on the flanking His residues [59].

However, the requirement for negatively charged headgroups does not appear to be absolute, although our understanding is not yet complete. Liposomes made entirely of zwitterionic lipids (usually PC) are not bound by most dehydrins, and the binding is attenuated in high ionic strength solutions [55,56,60], which does suggest the importance of negative charges. Despite having an anionic charge, liposomes made of pure PI do not bind dehydrins [52]; one explanation for this could be steric hindrance by the carbohydrate headgroup [61]. Supporting this is the observation that ZmDHN1 can bind PC:PI vesicles where the headgroups are more spread out, allowing access to the anionic phosphate [52]. Furthermore, surface plasmon resonance (SPR) has detected a weak interaction between Lti30 and PC liposomes [62]. Whether this is a testament to the sensitivity of SPR or a specific feature of the Lti30 dehydrin is unclear [61]. However, it has been suggested that the interactions made with PC vesicles are charge-based, as the ζ-potential of a PC membrane can be non-zero depending on its packing [58,63].

Several studies have shown how dehydrins may protect the membrane. Dehydrins have been shown to lower the transition temperature (T_m_) of various membrane compositions as well as lower the relative humidity at which the H_II_ phase transition occurs [55,59,62]. Lowering the Tm would allow the membrane to maintain its fluidity at a lower environmental temperature, thereby allowing it to function normally despite the cold while preventing the transition to the HII phase. While dehydrins have been shown to affect membrane fusion, the effect appears to be dependent on the length of the protein. In the case of *V. riparia* K_2_, the protein was able to prevent the fusion of two liposomes, thereby preventing the exchange of membranes. This was determined by measuring the diameter of PC:PA liposomes before and after freezing using dynamic light scattering (DLS). An increasing concentration of K_2_ was shown to reduce the size of the fused liposomes after freeze/thaw treatment, while PEG3350, which has a similar hydrodynamic radius to K_2_, was unable to prevent fusion [55]. This, along with the fact that deletion of the ϕ-segment did not impair the ability of the K-segments to prevent membrane fusion, suggested that membrane fusion is not a result of a molecular shield-like effect [55,64]. Instead, dehydrins may be preventing the liposomes from coming too close together and fusing, as well as hydrating the surface of the membrane due to their high propensity for water-binding and ability to prevent the water from freezing [55,65].

The ability of Lti30 to prevent membrane fusion has not been reported. However, this dehydrin does have the unusual ability to cluster liposomes when the His-dipeptides are protonated, which may be a consequence of the relatively large size of the dehydrin; the six K-segments could facilitate the binding of several liposomes at once [56]. The authors propose that the amphipathic helix may be forming hydrophobic interactions with one membrane and electrostatic interactions with another. It was later found that Lti30 is able to maintain a more consistent inter-bilayer distance between lamellar membranes as water content increases, as compared to the protein-free membranes which swell more [59]. This was speculated to be a result of the K-segments binding one bilayer while the ϕ-segments either extend away from the membrane or return, which then determines whether the next K-segment binds a different bilayer or the same one. They propose that the longer ϕ-segments are more likely to extend outwards, allowing the following K-segment to bind to another bilayer, anchoring them together and providing the necessary structure to maintain membrane integrity and avoid rupture as water content varies. This has been corroborated by SAXS experiments that found that the maximum distance between bilayers bound by Lti30 was consistent with the length of the fully extended long φ-segments [59].

## 6. Protein Cryoprotection

Dehydrins have been implicated in protein cryoprotection from freezing stress in many studies [41,50,66,67,68,69,70,71,72,73,74,75,76]. The most commonly used enzyme is lactate dehydrogenase (LDH), a cold-labile protein that denatures and loses almost all activity after freeze-thaw stress, although other enzymes such as malate dehydrogenase and alcohol dehydrogenase have been tested [50,60,77,78,79,80,81]. Several studies report the PD_50_ value, which is the concentration of dehydrin required to restore 50% of the enzyme’s activity, and can hence be considered a measure of the protective efficiency of a dehydrin. The main result across all of these studies is that increasing concentrations of dehydrins are able to provide increasing protection for enzyme activity; with a sufficiently high concentration, full enzymatic activity can be restored.

There has been considerable interest in the field when it comes to trying to understand what role the conserved segments may have in protecting LDH from freeze/thaw damage. There have been reports of the K-segment (and more recently, the F-segment) being involved in cryoprotection of LDH by using 15-residue peptides of these segments in the freeze/thaw assay [73,76]. Intriguingly, in the case of both motifs the hydrophobic residues were shown to play an essential role in this cryoprotective effect. The hydrophobic residues were determined to form solvent-exposed hydrophobic patches (four residues splayed out in different directions in the F-segment, or an amphipathic helix in the K-segment) using PEP-FOLD3 [73,76]. The authors proposed that these hydrophobic patches may then intercalate between LDH molecules that are beginning to denature, transiently interact with the hydrophobic regions, and keep the LDH from aggregating, which allows the enzyme surface to undergo ‘hydrophobic hydration’ [76].

Several other studies suggest that the exact sequence of the K-segment is not critical to enzyme cryoprotection. The first piece of evidence comes from NMR studies. Specific interactions between dehydrins and LDH do not appear to be a part of the cryoprotective effect, as NMR experiments detected no direct interactions between K_2_ from *V. riparia* and LDH [71]. The second discrepancy comes from the difference in cryoprotection offered by the same K_2_ protein, the KK-peptide and the K-peptide, the latter two of which lack the ϕ-segment. If K-segments were important, one would expect that the PD_50_ value of the K_2_ protein and the KK-peptide to be essentially the same, and the K-peptide to be about twice that (i.e., higher values indicate lower efficiency). Instead, the KK-peptide has a PD_50_ value twice that of the K_2_ dehydrin and the K-peptide has a value nearly four times higher; thus, the ϕ-segment does contribute to protection. One explanation is that the increased hydrodynamic radius (R_h_) provided by the ϕ-segment is important [71]. These ideas are supported by a study involving K_n_ concatemers (K_2_ repeated with the ϕ-segment separating each), which are naturally large dehydrins, and the disordered polymer polyethylene glycol (PEG) [71]. These experiments showed that the longer the dehydrin (whether a natural protein or a concatemer) and the longer the PEG molecule, the more efficient the resulting enzyme protection (i.e., the lower the PD_50_ value). Furthermore, the full-length YSK_2_ dehydrin and its scrambled counterpart, ScrYSK_2_ (with the same amino acid composition in a random order) were both found to be similarly disordered and to have similar PD_50_ values [75]. The lack of specific interaction and reliance on R_h_ supports the idea that a molecular shield and/or volume exclusion effect may be taking place where the dehydrins and PEG may discourage unfolding by decreasing the available space and prevent aggregation by existing in the space between proteins, making highly transient, non-specific interactions [64].

However, this theory can only go so far to explain the cryoprotective role of dehydrins. The Frost protein from *Drosophila* is an example of an intrinsically disordered protein that, despite having an R_h_ similar to that of OpsDHN1, is unable to restore LDH activity to >20% of its initial activity after freeze-thaw, further implying that R_h_ is not the only factor in dehydrin cryoprotection [45]. While the PEG cryoprotection experiments show that the positive charge of the K-segments is not essential to the cryoprotective function, excessive charge in the Frost protein (net charge of −53) suggests that too high a negative charge may be a problem. In addition, the relationship between PD_50_ and R_h_ becomes asymptotic at larger R_h_ values, suggesting that at a certain point, an increasing R_h_ has less effect on the protective effects of a dehydrin. This may in part be explained by the notion that the protective effect of a crowder peaks when its R_h_ is similar to that of the molecule being protected, whereas at greater R_h_ values pockets between crowders can be created which trap multiple smaller enzymes [82].

A clue that may be able to reconcile these discrepancies in the cryoprotective assays could be the effects of dehydrins on water. Ferreira et al. [83] used the solvatochromic comparison method to determine the ability of various K-concatemers (K_2_, K_4_ and K_10_), HSPB6, elastin-like polypeptide (ELP), and PEG (1000, 8000 and 10,000) to participate in dipole–dipole interactions (π *) and hydrogen bonds as both donors (β) and acceptors (α) [84,85,86]. These coefficients were then used to determine isochemical properties (*a*, *b* and *z*) unique to each solute, which, when plotted in 3D, revealed a plane that was altered only slightly by the addition of PEG. The distances between the coordinates of the proteins relative to K_2_ were then plotted against PD_50_ and the logarithm of R_h_. Once again, the coordinates formed a plane, suggesting that the solvent properties of these intrinsically disordered proteins may contribute to the observed relationship between PD_50_ and R_h_. The authors suggest that as R_h_ increases, the effects that the dehydrins have on water become increasingly significant and begin to offset the minimizing effect that R_h_ has on PD_50_ [83].

## 7. DNA Binding and Protection

A review [61] reported that many localization studies often found dehydrins in the cytoplasm and nucleus. The Y-segment-containing dehydrins Y_n_SK_n_ and Y_n_K_n_, in particular, are frequently found in the nucleus, though dehydrins belonging to each sequence architecture group have been found there as well [77,78,87,88,89,90,91]. The localization of these proteins to the nucleus suggests that they may have a protective role there. This was first examined by Hara et al. [92], who employed electrophoretic mobility shift assays (EMSA) to test the ability of a K_n_S dehydrin, CuCOR15, to bind to a 200 bp fragment of DNA. No shifts were observed until Zn^2+^ was added, and no other divalent cation was able to facilitate this shift [92], suggesting that the complex was a specific interaction. Histidine dipeptides are likely involved in the Zn^2+^-dependent binding, as they were found to be important in the ability of CuCOR15 to bind Zn^2+^ [92,93]. Likewise, a Y_2_K dehydrin from *Vigna radiata* was unable to bind a 0.7 or 1 kbp DNA fragment until Zn^2+^ or Ni^2+^ was present [89]. However, shifts were observed in the absence of Zn^2+^ when DNA of 3 or 4 kbp were used [89], suggesting that a metal cation may only be required to bind to shorter oligonucleotides. In addition, Hara et al. [92] split CuCOR15 into five segments to determine which region(s) of the CuCOR15 are important for DNA binding. They found that rather than any of the identified conserved segments, the regions involved in DNA binding were two motifs unique to that dehydrin: a His-rich region and a polylysine segment.

Another study with the YSK_2_ type dehydrin from *Vitis riparia*, VrDHN1 (which has no His dipeptides), showed that Zn^2+^ does not improve binding, instead competing with DNA to bind VrDHN1 [22]. Interestingly, the K-segments of CuCOR15, which have lower affinity for DNA, are not as Lys-rich as those of most dehydrins [92]. The implication that Lys is important in these interactions was strengthened by the results of additional experiments [22] which revealed that many of the interactions made with DNA involved Lys residues within the K-segments. Variable shifts indicated that while individual proteins may have been forming interactions using different residues, the greatest shifts often belonged to residues separated by about ten amino acids, which would put them in line with the DNA backbone after each full helical turn. Thus, a model wherein dehydrins line up parallel to the main axis of DNA was proposed. This would lead to the potential number of bound dehydrins being proportional to the length of the DNA. As a result, more interactions could be made with longer DNA, increasing the total binding strength of an otherwise low-affinity interaction. A randomly scrambled version of YSK_2_ (i.e., with the same amino acid composition in a different order) was unable to bind with the same efficiency as the unscrambled YSK_2_, further implicating the K-segment in this interaction [22]. The same study showed that the dehydrin–DNA interaction is nonspecific, and could in theory protect any DNA sequence. A non-specific interaction was observed using the bacterial pkTOL2 plasmid, indicated by a shift that increased proportional to the rising concentration of dehydrin used. In contrast, a specific interaction would have produced discrete shifts, and no further shift would have been observed once all binding sites were occupied [22].

## 8. Conclusions

**Figure 1:**
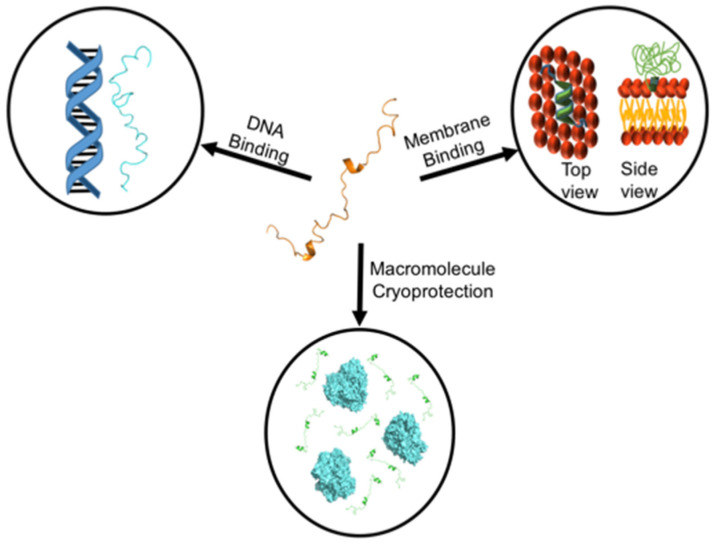
In vitro evidence suggests that dehydrins are involved in the binding and stabilization of DNA and membranes, as well as in the cryoprotection of proteins. Depicted here are representations of the interactions thought to take place in these scenarios. The K_2_ dehydrin from *Vitis riparia* is shown in different conformations that it samples (alone in the center, extended and parallel to DNA, and acting as a crowder around lactate dehydrogenase).

Here, we provide a summary of the discoveries pertaining to the biochemical function of dehydrins. These studies show that dehydrins are involved in multiple protective functions on very distinct biomacromolecules, including membrane lipids, proteins, and DNA (Figure 1). This is not surprising for an intrinsically disordered protein, as their ability to be highly flexible and sample diverse structural states has been correlated with their ability to carry out multiple functions, an effect sometimes termed ‘moonlighting’. A considerable amount of work has been carried out in order to better understand the significance of the various conserved sequence motifs, in addition to finding potential binding partners and theorizing about the interactions that might be made. Ideally, future advancements in dehydrin research will focus on finding direct evidence of the interactions that dehydrins are proposed to make. Additionally, a pivot away from in vitro experiments and towards studies focused on the ways in which dehydrins behave in the cell will be crucial if we are to truly understand the molecular mechanisms by which dehydrins function.

## Figures and Tables

**Table 1:** The effects of dehydrin expression on plant stress response.

Plant/Author	LEA Protein	Expression System	Applied Stress	Observed Phenotype	Post-Stress Change without Dehydrin	Post-Stress Change with Dehydrin
*Vigna* *Unguiculata* (L.) Walp. [11]	Unnamed Dehydrin: Present in line 1393-2-11	Naturally occurring	Seeds grown for 27 days at 15 °C	Percent emergence	13%	50%
			Seeds incubated for 12 h at 15 °C	Electrolyte leakage	41.3 mS/m·g	27.1 mS/m·g
*Oryza sativa* L. [12]	PMA80, wheat dehydrin	pGHNC5, Act1 promoter	Six-week old plants grown with 200 mM NaCl for nine days	Electrolyte leakage from leaves	+342% from day 3–7	+155% from day 3–7
				Height of plant after recovery	48.2 ± 2.8 cm	63.1 cm
				Fresh/dry shoot weight after recovery	Fresh: 3.37 g Dry: 0.67 g	Fresh: 4.8 g Dry: 1.025 g
			Six-week old plants denied water for eight days	Height of plant, after eight days of recovery	47.8 ± 2.4 cm	63.6 cm
				Fresh/dry shoot weight, after eight days of recovery	Fresh 3.23 ± 0.06 g Dry: 0.7 ± 0.01 g	Fresh: 4.8 g Dry: 1.053 g
*Nicotiana tabacum* L. [13]	*Citrus unshiu* dehydrin (CuCOR19)	pBI121, CaMV 35S promoter	Seedlings incubated at −4 °C for three hours	Electrolyte leakage expressed as percent of total	19.2 ± 2.6%	12.3 ± 2.6%
				MDA production	4.6 nmol/g fresh weight	2.7 nmol/g fresh weight
			Grown at 15 °C	Germination	72% (GD_50_ = 11.9 days)	88% (GD_50_ = 8.1 days)
				Increase in seedling fresh weight over 15 days (day 6 to 21 after sowing)	3.9 mg/plant	6.7 mg/plant
*Nicotiana tabacum* NC89 [14]	*Brassica juncea* dehydrins (BjDHN2/BjDHN3)	pBI121, CaMV 35S promoter	Leaf disks from four-week old plants floated on MS (⅛) solution at 4 °C for seven days	Chlorophyll content	43% mg/g of dry weight	BjDHN2: 60% mg/g·DW BjDHN3: 69% mg/g·DW
			Grown with 150 mM NaCl for 10 days followed by two weeks of recovery	Relative water content	75%	BjDHN2: 92% BjDHN3: 90%
				Electrolyte leakage	16%	BjDHN2: 3% BjDHN3: 7%
				MDA content	4.75 mmol/g of fresh weight	BjDHN2 3.5 mmol/g·FW BjDHN3: 3.5 mmol/g·FW
*Nicotiana tabacum* L. cv NC89 [15]	Maize dehydrin, ZmDHN2b	pBI121, CaMV 35S promoter	Germinated at 15 °C	Germination	Began germinating on day 11. By day 30 <60% had germinated.	Began germinating on day 7. By day 30, 80% had germinated.
			Germinated and grown at 25 °C for four days. Incubated at 15 °C for two weeks.	Root length	<1.0 cm	>1.8 cm
			Six-week old plants incubated at 4 °C for 24 h	MDA content	2.5-fold increase over 24 h	Two-fold increase over 24 h
				Electrolyte leakage	3.14-fold increase over 24 h	2.3-fold increase over 24 h
*Nicotiana tabacum* [16]	*Prunus mume* dehydrins (PmLEA10, PmLEA19, PmLEA20, PmLEA29)	pEarleyGate203, CaMV 35S promoter	Two-month old plants incubated at 4 °C for 24 h	Increase in electrolyte leakage relative to untreated plants	1.9%	PmLEA10: 1.3% PmLEA19: 1.15% PmLEA20: 0.75% PmLEA29: 1.2%
				Increase in MDA content relative to untreated plants	3.3%	PmLEA10: 1.6% PmLEA19: 1.8% PmLEA20: 0.75% PmLEA29: 1.9%
			Two-month old plants denied water for 15 days	Increase in electrolyte leakage relative to untreated plants	1.5%	PmLEA10: 1.3% PmLEA19: 1.15% PmLEA20: 0.8% PmLEA29: 1.2%
				Increase in MDA content relative to untreated plants	2%	PmLEA10: 1.5% PmLEA19: 1.8% PmLEA20: 0.75% PmLEA29: 1.25%

## Data Availability

Not applicable.
